# Sex Difference in the Association of Osteoporosis and Osteopenia Prevalence in Patients with Adrenal Adenoma and Different Degrees of Cortisol Excess

**DOI:** 10.1155/2022/5009395

**Published:** 2022-03-18

**Authors:** Shoichiro Izawa, Kazuhisa Matsumoto, Kazuhiko Matsuzawa, Takuyuki Katabami, Takanobu Yoshimoto, Michio Otsuki, Masakatsu Sone, Yoshiyu Takeda, Shintaro Okamura, Takamasa Ichijo, Mika Tsuiki, Tomoko Suzuki, Mitsuhide Naruse, Akiyo Tanabe

**Affiliations:** ^1^Division of Endocrinology and Metabolism, Tottori University Faculty of Medicine, Yonago, Japan; ^2^Division of Metabolism and Endocrinology, Department of Internal Medicine, St. Marianna University School of Medicine, Yokohama City Seibu Hospital, Yokohama, Japan; ^3^Department of Molecular Endocrinology and Metabolism, Tokyo Medical and Dental University, Tokyo, Japan; ^4^Department of Metabolic Medicine, Osaka University Graduate School of Medicine, Osaka, Japan; ^5^Department of Endocrinology, Tokyo Women's Medical University, Tokyo, Japan; ^6^Department of Diabetes, Endocrinology and Nutrition, Kyoto University, Kyoto, Japan; ^7^Division of Metabolism and Endocrinology, Department of Internal Medicine, St. Marianna University School of Medicine, Kawasaki, Japan; ^8^Department of Internal Medicine, Graduate School of Medical Science, Kanazawa University, Kanazawa, Japan; ^9^Department of Endocrinology, Tenri Hospital, Tenri, Japan; ^10^Department of Diabetes and Endocrinology, Saiseikai Yokohamashi Tobu Hospital, Yokohama, Japan; ^11^Department of Endocrinology and Metabolism, National Hospital Organization Kyoto Medical Center, Kyoto, Japan; ^12^Department of Public Health, International University of Health and Welfare School of Medicine, Narita, Japan; ^13^Clinical Research Institute of Endocrinology and Metabolism, National Hospital Organization Kyoto Medical Center, Kyoto, Japan; ^14^Endocrine Center, Ijinkai Takeda General Hospital, Kyoto, Japan; ^15^Division of Endocrinology, National Center for Global Health and Medicine, Tokyo, Japan

## Abstract

**Objective:**

Osteoporosis and osteopenia (OS/OP) are frequent in patients with adrenal adenomas associated with cortisol excess (CE). However, the relationship between OS/OP and CE severity considering sex differences is unknown.

**Design:**

A cross-sectional observational study from January 2006 to December 2015. *Patients*. 237 patients with adrenal adenoma associated with CE, including Cushing's syndrome and mild autonomous cortisol secretion (MACS), diagnosed in 10 referral centers in Japan. MACS was defined by 1 mg overnight dexamethasone suppression test (DST) cortisol level >1.8 *μ*g/dL. *Measurements*. Prevalence of fragility fractures, medication for osteoporosis, and bone mineral density.

**Results:**

In total, 112 of 237 patients, who were predominantly female (*P* < 0.001) and had lower BMI (*P*=0.013), had OS/OP. Patients with OS/OP was significantly affected by CE (*P* < 0.01) than those without. The adjusted odds ratio (OR) for predicting OS/OP was obtained in multivariate logistic regression analysis. Clinical measures of CE, 1 mg DST cortisol levels, were positively associated with OS/OP in total cases (OR 1.124, 95% CI: 1.070–1.181, *P* < 0.001) and the cases with MACS (OR 1.156, 95%CI: 1.046–1.278, *P*=0.005). A cutoff value of 1 mg DST cortisol level >5.0 *μ*g/dL was associated with OS/OP differently between men and women. OS/OP risk in men with MACS was significantly affected only by 1 mg DST cortisol levels. However, OS/OP risk in women with MACS was significantly affected by 1 mg DST cortisol levels and age.

**Conclusions:**

CE severity in adrenal adenoma is positively associated with OS/OP. However, the associated factors of OS/OP in the patients with MACS are different between men and women.

## 1. Introduction

Cushing's syndrome (CS) includes several subtypes of disease characterized by cortisol excess (CE). ACTH-independent CS consists of adrenal and iatrogenic CS. Adrenal CS is typically caused by CE originating from adrenocortical neoplasms, and the production of ACTH is suppressed by excess cortisol [1, 2]. Suppressed ACTH also decreases the endogenous production of adrenal androgens [3, 4]. Iatrogenic CS is induced by the therapeutic administration of synthetic glucocorticoids, causing suppression of endogenous ACTH and adrenal androgens. In contrast, ACTH-dependent CS, including Cushing's disease due to pituitary neoplasm and ectopic ACTH-producing tumors, is caused by excess ACTH. Adrenal androgens are also excessively produced in addition to the CE [3, 4].

Osteoporosis is one of the well-known complications of CS. Approximately, 21% of patients with CS experience a fragility fracture before diagnosis [1]. However, the prevalence of osteoporosis and its association with endocrinological severity have previously been discussed mainly in patients with CS including various subtypes [1, 5, 6].

The clinical background of ACTH-independent and -dependent CS differs with regard to adrenal androgens [3]. Endogenous adrenal androgen is associated with the activation of osteoblasts or bone turnover [7, 8]. The osteoporosis seen in adrenal CS has been speculated to be more influenced by CE, especially in women.

Recently, mild autonomous cortisol secretion (MACS), defined by a 1 mg overnight dexamethasone suppression test (1 mg DST) cortisol level >1.8 *μ*g/dL but without the classical symptoms and signs of overt CS, has been shown to be prevalent, affecting up to 45% of patients with adrenal adenomas [9]. The indication for adrenalectomy in MACS is decided based on the severity of CE and coexistent complications, including glucose impairment, hypertension, obesity, and dyslipidemia. In diagnostic guidelines published by the Japan Endocrine Society, a 1 mg DST cortisol level >5.0 *μ*g/dL is an indication for adrenalectomy. Patients with a 1 mg DST cortisol level of 3.0–5.0 *μ*g/dL are recommended adrenalectomy based on the presence of complications [10]. In a cross-sectional study with prospective enrollment, MACS was associated with a reduction in osteocyte function and number, with restoration after adrenalectomy [11]. These data suggest that relatively mild ACTH-independent CE can be a risk factor for osteoporosis. However, how the severity of CE in adrenal adenoma differently affects osteoporosis and osteopenia (OS/OP) by sex has not been demonstrated at all.

In the present study, we conducted a retrospective cross-sectional analysis to evaluate the sex differences in association between the severity of CE and OS/OP in patients with adrenal adenoma.

## 2. Materials and Methods

### 2.1. Study Populations

This retrospective observational study was conducted as a part of the Advancing Care and Pathogenesis of Intractable Adrenal Disease in Japan (ACPA-J) study, which was based on a registration system for a cohort of patients with adrenal tumors. Patients aged 20 to 90 who were diagnosed with MACS and overt CS originated from adrenal adenoma between January 2006 to December 2015 in 10 referral centers, including the National Center for Global Health and Medicine, National Hospital Organization Kyoto Medical Center, St. Marianna University Yokohama City Seibu Hospital, Tottori University Hospital, Tokyo Medical and Dental University, Osaka University Graduate School of Medicine, Kyoto University, Kanazawa University Graduate School of Medical Science, Tenri Hospital, and Saiseikai Yokohama-shi Tobu Hospital, were enrolled. Because all participating centers accept patients considered the indication of adrenalectomy, the proportion of overt CS was higher than previous reports enrolling adrenal incidentaloma [9, 12, 13]. Patients with ACTH-dependent CS and iatrogenic CS were not included in the study. Patients with MACS and overt CS originated from adrenocortical carcinoma and adrenocortical hyperplasia were excluded from the analysis. The patients' clinical characteristics, biochemical examination results, radiological findings, and pathological diagnoses were collected electronically using a web registry system. The present study was conducted using a dataset validated in April 2019.

The ACPA-J study was conducted in accordance with the Declaration of Helsinki and the guidelines for clinical studies published by the Ministry of Health, Labour, and Welfare, Japan. The study protocol was approved by the Ethics Committee of the National Center for Global Health and Medicine and each institutional ethics committee of the participating centers. This study was registered at UMIN ID 000021437.

### 2.2. Definition of Cortisol Excess

All participants were diagnosed on the basis of the diagnostic criteria for MACS published by Japan Endocrine Society and Cushingoid features [10], which were defined by the positivity of at least one sign, such as easy bruising, facial plethora, moon face, hirsutism, acne, central obesity, proximal myopathy, and striae. All patients with a 1 mg DST cortisol >1.8 *μ*g/dL were enrolled in this study [14]. Of 292 patients with confirmed adrenal adenoma in each institution, a total of 237 were included in this study. The reasons for excluding patients included a past history of adrenalectomy (*n* = 18), missing 1 mg DST data (*n* = 13), and insufficient data for OS/OP (*n* = 26).

### 2.3. Definition of Osteoporosis and Osteopenia

Bone mineral density (BMD) at the lumbar spine, femoral neck, and distal radius were measured using dual-energy X-ray absorptiometry using a Discovery system (Hologic Japan, Tokyo, Japan) in three centers, Horizon system (Hologic Japan, Tokyo, Japan) in two centers, QDR4500 system (Hologic Japan, Tokyo, Japan) in one center, PRODIGY system (GE Health Care Japan, Tokyo, Japan) in two centers, and iDXA system (GE Health Care Japan, Tokyo, Japan) in two centers. BMD was automatically calculated from the bone mineral content (in grams) and bone area (in square centimeters) and expressed as an absolute value in grams per square centimeter. The young adult mean (YAM) is the percentage given from the mean BMD for a healthy young adult (100%) reference population. The diagnosis of OS/OP was based on the criteria for primary osteoporosis published by the Japanese Society for Bone and Mineral Research and Japan Osteoporosis Society Joint Review Committee [15]. Osteoporosis was diagnosed based on a past history of fragility fracture, medication for osteoporosis, and less than 70% of the YAM, which is nearly equal to <−2.5 SD of the T-score in the World Health Organization criteria, obtained from the lumbar spine or femoral neck [15]. Osteopenia was defined as a value of 70%–80% of YAM, which corresponds to −2.5 SD < T-score <−1.7 to −1.8 SD, without a history of fragility fracture and medication for osteoporosis [15].

### 2.4. Biochemical Parameters

The estimated glomerular filtration rate (eGFR) was calculated using the following equation: eGFR (mL/min/1.73 m^2^) = 194 × serum creatinine (−1.094) × age (−0.287) × 0.739 (if female). Serum and urinary cortisol concentrations were evaluated using an electrochemiluminescence immunoassay (ECLIA) (Roche, Tokyo, Japan) at two centers, chemiluminescent immunoassay (CLIA) (Siemens Healthineers, Tokyo, Japan) at two centers, chemiluminescent immunoassay (CLEIA) (Siemens Healthineers, Tokyo, Japan) at one center, an enzyme immunoassay (EIA) (TOSOH, Tokyo, Japan) at three centers, and a CLEIA (Beckman Coulter, Tokyo, Japan) at two centers. Their coefficients of variation are <15%. The ranges of measurement of these kits are 0.054–63.4, 0.50–75.0, 1.0–50.0, 0.2–60.0, and 0.4–60.0 *μ*g/dL, respectively. Plasma ACTH concentrations were evaluated using an ECLIA (Roche, Tokyo, Japan) at seven centers, immunoradiometric assay (Mitsubishi Chemical, Tokyo, Japan) at one center, CLEIA (Siemens Healthineers, Tokyo, Japan) at one center, and EIA (TOSOH, Tokyo, Japan) at one center. Their coefficients of variation are <15%. The ranges of measurement of these kits are 1.00–2000, 5.0–1200, 10–1250, and 2.0–2000 pg/mL, respectively. Serum dehydroepiandrosterone-sulfate (DHEA-S) concentrations were evaluated using a CLEIA (Beckman Coulter, Tokyo, Japan) at eight centers, CLEIA (Siemens Healthineers, Tokyo, Japan) at one center, and CLIA (Siemens Healthineers, Tokyo, Japan) at one center. Their coefficients of variation are <15%. The ranges of measurement of these kits are 2.0–1000, 15–1000, and 3.0–1500 *μ*g/dL, respectively.

Plasma ACTH and serum cortisol were evaluated by supine position in the morning, which was between 6 and 10 AM, and night, which was between 9 and 11 PM. 1 mg DST was performed by usual fashion, in which 1 mg of dexamethasone was given at 11 PM and the level of serum cortisol was evaluated between 8 and 9 AM in the following morning.

### 2.5. Statistics

Statistical analysis was performed using the IBM SPSS software (version 24; IBM Corp., Tokyo, Japan). Continuous variables are expressed as a median and interquartile range. Categorical variables are presented as an actual number and percentages. Continuous variables were compared using the Mann–Whitney *U* test, and categorical variables were compared using the chi-square test. Based on 10 events per predictor rule, multivariate logistic regression analysis was performed after adjusting for variables including patient background and severity of CE. The association between CE and OS/OP was evaluated separately for as follows: 1 mg DST cortisol results considered as a dichotomous variable (>5.0 *µ*g/dL vs. 1.9–5.0 *µ*g/dL (used as the reference)), 1 mg DST cortisol levels considered as a continuous variable (*µ*g/dL), and the presence of Cushingoid features vs. without Cushingoid features (used as the reference). An odds ratio (OR) > 1 indicated an increased likelihood of OS/OP. Cutoff values of 1 mg DST cortisol levels for predicting OS/OP were also compared between male and female by receiver operator characteristic (ROC) curve analysis. All tests were two-tailed. Values of *P* < 0.05 were considered statistically significant.

## 3. Results

### 3.1. Osteoporosis and Osteopenia in the Patients with Adrenal Adenoma Associated with Cortisol Excess

A total of 237 patients with adrenal adenoma associated with CE were included in this study. The median age of the patients was 56 years, and 76.8% were female. Cushingoid features were present in 40.5% of participants. Two hundred three of the 237 enrolled patients (85.7%) presented unilateral adenoma. The median BMD at the femoral neck was at a level indicative of osteopenia. One hundred twelve of the 237 enrolled patients (47.2%) were defined as OS/OP. Eighty-nine of the 112 patients with OS/OP (79.4%) met the definition of osteoporosis (data not shown).

OS/OP was more prevalent in female (54.4%) than in male (23.6%) (*P* < 0.001). Presence of Cushingoid features was significantly higher in patients with OS/OP (*P* < 0.001). The body mass index (BMI) in patients with OS/OP was significantly smaller than that in patients without OS/OP (*P*=0.013). Size and laterality of adrenal adenoma, serum calcium, renal function, lipid profile, and glucose metabolism were not related to OS/OP status (Table 1).

On endocrinological evaluation, morning, night, and 1 mg DST cortisol levels and 24-hour urinary cortisol levels were significantly higher in patients with OS/OP than in those without (*P* < 0.01). Morning and night ACTH and DHEA-S levels were significantly lower in patients with OS/OP (*P* < 0.005). Positivity of 1 mg DST cortisol >5.0 *μ*g/dl was significantly higher in patients with OS/OP (*P* < 0.001) (Table 1). These results suggest that the severity of CE was related to the presence of OS/OP.

To investigate whether the severity of CE in total cases and the cases with MACS was positively associated with OS/OP, multivariate logistic regression analysis was used to evaluate the positivity of 1 mg DST cortisol level >5.0 *μ*g/dl (vs. 1 mg DST cortisol level of 1.9–5.0 *μ*g/dL), continuous variables of 1 mg DST levels (*µ*g/dL), and the positivity of Cushingoid features as shown in Table 2; 1 mg DST cortisol level >5.0 *μ*g/dl (OR 3.358, 95% CI: 1.630–6.917, *P* < 0.001), 1 mg DST cortisol levels (OR 1.124, 95% CI: 1.070–1.181, *P* < 0.001), and Cushingoid features (OR 3.924, 95% CI: 2.045–7.530, *P* < 0.001) were positively associated with OS/OP in total cases. 1 mg DST cortisol levels (OR 1.156, 95% CI: 1.046–1.278, *P*=0.005) was also positively associated with OS/OP in the cases with MACS. However, 1 mg DST cortisol level >5.0 *μ*g/dl was not positively associated with OS/OP in the cases with MACS (OR 2.187, 95% CI: 0.973–4.914, *P*=0.058). Female sex in the analysis of both total cases and the cases with MACS was also positively associated with OS/OP in the models using positivity of 1 mg DST cortisol level >5.0 *μ*g/dl, 1 mg DST cortisol levels and Cushingoid features. Continuous variables of age in the analysis of the cases with MACS was positively associated with OS/OP in the model of using positivity of 1 mg DST cortisol level >5.0 *μ*g/dl (OR 1.036, 95% CI: 1.001–1.072, *P*=0.042) and that of using 1 mg DST cortisol levels (OR 1.041, 95% CI: 1.005–1.078, *P*=0.024). Continuous valuables of BMI in the analysis of total cases was negatively associated with OS/OP in the model of using the positivity of Cushingoid features (OR 0.937, 95% CI: 0.878–0.999, *P*=0.047).

### 3.2. Sex Differences in Association with the Prevalence of Osteoporosis and Osteopenia

The prevalence of OS/OP was compared between men and women in total cases by univariate analysis (Table 3). Male patients with a 1 mg DST cortisol level >5.0 *μ*g/dL relative to those with a 1 mg DST cortisol level of 1.9–5.0 *μ*g/dL (OR 16.000, 95% CI: 1.902–134.580, *P*=0.003), higher 1 mg DST cortisol levels (*μ*g/dL) (OR 1.150, 95% CI: 1.039–1.273, *P*=0.007), and Cushingoid features (OR 6.343, 95% CI: 1.509–26.656, *P*=0.014) had a greater increase in the prevalence of OS/OP than female patients with a 1 mg DST cortisol level >5.0 *μ*g/dL relative to those with a 1 mg DST cortisol level of 1.9–5.0 *μ*g/dL (OR 2.190, 95% CI: 1.038–4.619, *P*=0.041), higher 1 mg DST cortisol levels (*μ*g/dL) (OR 1.092, 95% CI: 1.043–1.144, *P* < 0.001), and Cushingoid features (OR 2.701, 95% CI: 1.472–4.958, *P*=0.002). The continuous variables of age and BMI were not associated with the prevalence of OS/OP in both men and women.

The prevalence of OS/OP was also compared between men and women with MACS. Male patients with a 1 mg DST cortisol level >5.0 *μ*g/dL relative to those with a 1 mg DST cortisol level of 1.9–5.0 *μ*g/dL (OR 11.077, 95% CI: 1.201–102.193, *P*=0.013) and higher 1 mg DST cortisol levels (*μ*g/dL) (OR 1.221, 95% CI: 1.027–1.452, *P*=0.024) were significantly associated with the prevalence of OS/OP. However, the prevalence of OS/OP in female patients with MACS was not associated with a 1 mg DST cortisol level >5.0 *μ*g/dL relative to those with a 1 mg DST cortisol level of 1.9–5.0 *μ*g/dL (OR 1.340, 95% CI: 0.583–3.084, *P*=0.533) and higher 1 mg DST cortisol levels (*μ*g/dL) (OR 1.092, 95% CI: 0.983–1.213, *P*=0.100). The higher age (OR 1.044, 95% CI: 1.006–1.084, *P*=0.024) was only associated with the prevalence of OS/OP in female patients with MACS (Table 3).

Multivariate logistic regression analysis was performed to compare the associated factors of OS/OP in female patients. CE using the model of 1 mg DST cortisol level >5.0 *μ*g/dL relative to 1 mg DST cortisol level of 1.9–5.0 *μ*g/dL (OR 2.561, 95% CI: 1.142–5.742, *P*=0.022), higher 1 mg DST cortisol levels (OR 1.124, 95% CI: 1.063–1.189, *P* < 0.001), and presence of Cushingoid features (OR 3.833, 95%CI: 1.829–8.030, *P* < 0.001) was positively associated with OS/OP in total female patients. Using 1 mg DST cortisol levels as continuous variable, higher age (OR 1.031, 95%CI: 1.004–1.059, *P*=0.027) was also positively associated with OS/OP (Table 4). On the other hand, the prevalence of OS/OP in the cases with MACS was significantly affected by age (OR 1.041, 95% CI: 1.002–1.083, *P*=0.041); however, positivity of 1 mg DST cortisol level >5.0 *μ*g/dL relative to 1 mg DST cortisol level of 1.9–5.0 *μ*g/dL (OR 1.686, 95% CI: 0.687–4.140, *P*=0.254) had no significant relationship. Using 1 mg DST cortisol levels as continuous variables, OS/OP in the cases with MACS was significantly affected by higher 1 mg DST cortisol levels (OR 1.129, 95% CI: 1.005–1.267, *P*=0.041) and higher age (OR 1.047, 95% CI: 1.006–1.090, *P*=0.024).

ROC analysis for predicting OS/OP by 1 mg DST cortisol levels was compared between male and female. The area under the curve (AUC) in total male patients (AUC: 0.780 ± 0.069, 95% CI: 0.644–0.916, *P* < 0.001) and male patients with MACS (AUC: 0.801 ± 0.098, 95% CI: 0.610–0.992, *P*=0.012) were higher than total female patients (AUC: 0.665 ± 0.041, 95% CI: 0.585–0.745, *P* < 0.001) and female patients with MACS (AUC: 0.580 ± 0.058, 95% CI: 0.466–0.693, *P*=0.170). The analysis for female patients with MACS was not significant for predicting OS/OP (Table 5).

## 4. Discussion

In the present study, we demonstrated that the severity of CE was positively associated with developing OS/OP. Presence of Cushingoid features and biochemical evaluation using 1 mg DST cortisol, morning cortisol, night cortisol, 24-hour urinary cortisol, and DHEA-S levels contributed to the prediction of OS/OP. Multivariate logistic regression analysis demonstrated that positivity of 1 mg DST cortisol level >5.0 *μ*g/dl and 1 mg DST cortisol levels were positively associated with OS/OP in adrenal adenoma associated with CE. A cutoff value for differentiating apparent CE of 1 mg DST cortisol level >5.0 *μ*g/dL [10, 14] was consistent with predicting OS/OP in both sexes. However, the influence of CE was smaller in women. The prevalence of OS/OP in women was also affected by age especially in the cases with MACS.

Approximately, 47% of our cohort had OS/OP at time of diagnosis with adrenal adenoma associated with CE. In the previous study, the prevalence of vertebral fractures was 21% in patients with CS including Cushing's disease and ectopic ACTH-producing tumors [1]. However, approximately 46%–83% of patients with adrenal adenoma associated with CE had vertebral fractures although Cushingoid features were absent and they presented with mild CE [16]. There is a possibility that the severity of OS/OP in patients with adrenal adenoma associated with CE is not only dependent on CE but also other unknown factors.

In a study comparing adrenal CS and Cushing's disease [3], the prevalence of osteoporosis was significantly high in patients with adrenal CS. The authors speculated that adrenal androgen was downregulated by the suppression of ACTH in adrenal CS [17]. Tauchmanova et al. reported that vertebral fractures were common in female patients with CE regardless of the severity [18]. Adrenal androgen plays an important role in osteoblast activation, bone mass, and bone quality, especially in women [4, 7, 8, 19, 20]. In the present study, not including patients with ACTH-dependent CS, CE, and female sex were positively associated with developing OS/OP (Table 2). The suppression of ACTH by CE may contribute to the downregulation of adrenal androgen, which can have a negative effect on bone metabolism, especially in women.

OS/OP was diagnosed based on the existence of fragility fractures, medication use, and BMD as assessed using the YAM, which was a percentage calculated in comparison with the mean BMD obtained from young adults, in the present study. BMD negatively correlates with age in general [15]. BMD in postmenopausal women decreases dramatically with age in contrast to in premenopausal women [21–23]. Multivariate logistic regression analysis evaluating 1 mg DST cortisol demonstrated that OS/OP was affected by age in female patients with MACS. Female sex was positively associated with OS/OP evaluated using 1 mg DST cortisol in total cases and the cases with MACS (Table 2).

Our study demonstrated that, in total patients and male patients with MACS, CE was positively associated with OS/OP (Tables 3 and 4). Chiodini et al. demonstrated that the coexistence of adrenal incidentaloma and MACS increased the incidence of vertebral fractures and low BMD in eugonadal male patients [24]. Male patients can be directly affected by CE. Female patients including overt CS can be affected CE and consequent downregulation of adrenal androgen [18]. However, female patients with MACS can be affected not only by CE. Aging can also be associated with OS/OP as in primary osteoporosis [15, 17, 21, 23, 25].

The indications for adrenalectomy for adrenal adenoma associated with CE are based on the diagnosis of overt adrenal CS, 1 mg DST cortisol levels, and severity of complications related to CE in the current guidelines [10, 14]. A guideline published by the European Society of Endocrinology and European Network for the Study of Adrenal Tumors (ENS@T), states that autonomous cortisol secretion with a 1 mg DST cortisol level >5.0 *μ*g/dL is an indication of adrenalectomy; a 1 mg DST cortisol level of 1.9–5.0 *μ*g/dL is defined as possible autonomous cortisol secretion. Adrenalectomy is considered after adding other clinical manifestations, including 24-hour urinary cortisol and metabolic disorders to possible autonomous cortisol secretion [14]. Other guidelines including those published by the Japan Endocrine Society provided similar criteria for adrenalectomy [10]. For considering adrenalectomy concerning fractures and decreased BMD, MACS defined by a 1 mg DST cortisol level >3.0 *μ*g/dL was used in several studies [17, 18, 26]. Morelli et al. reported that a criterion for detecting incidental vertebral fractures on adrenal incidentaloma was a 1 mg DST cortisol level >2.0 *μ*g/dL [27]. Several studies have demonstrated that 1 mg DST cortisol levels >1.8 *μ*g/dL can reduce bone quality [11, 28–30].

We calculated the OR among those with 1 mg DST cortisol levels >5.0 *μ*g/dL relative to those with 1 mg DST cortisol levels of 1.9–5.0 *μ*g/dL. The OR for OS/OP was significant for both sexes. However, the value of the OR was remarkably higher in male patients than in female patients. Furthermore, 1 mg DST cortisol levels and age were positively associated with OS/OP in female patients with MACS (Tables 3 and 5). These results suggested that the indication for adrenalectomy based on OS/OP should be determined by considering CE, sex and age. Compared with male patients, the deleterious effect to OS/OP may be exerted by milder CE in female patients with MACS.

The present study had some limitations. First, the evaluation of bone metabolic biomarkers was not performed [21]. The precise mechanism underlying the progression of osteoporosis, including bone quality, was not clearly demonstrated [28]. Second, the method for evaluating endocrinological variables and BMD differed between institutions. BMD was evaluated following Japanese criteria [15]. We enrolled patients with OS/OP, which is equivalent to a T-score <−1.7 to −1.8 SD in the World Health Organization criteria. Third, this was a retrospective and cross-sectional study with long enrolling period. The number of overt CS was higher than the previous studies evaluating adrenal incidentaloma [9, 12, 13]. Then, the prevalence of OS/OP can be different from the studies evaluating MACS and nonfunctioning adrenal adenoma. The effect of CE may be weakened by the analysis not comparing with nonfunctioning adrenal adenoma. Further studies with a unified evaluation and prospective design are warranted to confirm our results.

In conclusion, we demonstrated that the severity of cortisol excess associated with adrenal adenoma was independently associated with the coexistence of osteoporosis and osteopenia. Our data suggest that cortisol excess in mild autonomous cortisol secretion had different effects on osteoporosis and osteopenia by sex.

## Figures and Tables

**Table 1:** Clinical background and cortisol excess in patients with or without osteoporosis and osteopenia.

	Total (*n* = 237)	With OS/OP (*n* = 112)	Without OS/OP (*n* = 125)	*P* value
Age, yrs. (IQR)	56 (44–64)	57 (42–64)	55 (46–63)	0.494
Female, *n* (%)	182 (76.8)	99 (88.4)	83 (66.4)	<0.001^*∗*^
Cushingoid features, *n* (%)	96 (40.5)	62 (55.4)	34 (27.2)	<0.001^*∗*^
BMI, kg/m^2^ (IQR)	23.4 (21.4–26.7)	23.0 (20.8–26.0)	24.2 (22.1–27.2)	0.013^*∗*^
Unilateral adenoma, *n* (%)	203 (85.7)	97 (86.6)	106 (84.8)	0.346
Maximum tumor diameter, mm (IQR)	25 (21–30)	26 (21–31)	25 (20–29)	0.157
sCr, mg/dL (IQR)	0.63 (0.55–0.78)	0.63 (0.53–0.72)	0.64 (0.56–0.80)	0.742
eGFR, mL/min/1.73 m^2^ (IQR)	80.2 (65.5–95.4)	78.2 (64.7–96.3)	80.3 (65.9–90.7)	0.937
Ca, mg/dL (IQR)	9.1 (8.8–9.5)	9.1 (8.7–9.4)	9.2 (8.9–9.5)	0.098
TCH, mg/dL (IQR)	206 (178–231)	203 (176–235)	208 (179–229)	0.939
HDL-C, mg/dL (IQR)	59 (49–73)	60 (52–76)	59 (48–71)	0.465
LDL-C, mg/dL (IQR)	120 (100–146)	121 (95–148)	117 (100–140)	0.778
TG, mg/dL (IQR)	110 (81–152)	107 (80–151)	114 (83–154)	0.258
HbA1c, % (IQR)	5.8 (5.5–6.6)	5.9 (5.5–6.8)	5.8 (5.4–6.5)	0.796
PG, mg/dL (IQR)	92 (84–106)	90 (83–102)	97 (86–109)	0.068
Fragility fracture, *n* (%)	35 (14.8)	35 (31.3)	0 (0)	<0.001^*∗*^
Medication for osteoporosis, *n* (%)	25 (10.5)	25 (22.3)	0 (0)	<0.001^*∗*^
BMD lumbar spine YAM, % (IQR)	80 (70–95)	74 (68–81)	96 (91–105)	<0.001^*∗*^
BMD femoral neck YAM, % (IQR)	78 (71–89)	75 (68–79)	94 (86–100)	<0.001^*∗*^
BMD distal radius YAM, % (IQR)	93 (84–104)	93 (77–104)	97 (89–106)	0.934
Morning ACTH, pg/mL (IQR)	5.0 (2.0–9.0)	2.3 (1.0–5.0)	6.0 (3.8–11.8)	<0.001^*∗*^
Morning cortisol, *µ*g/dL (IQR)	13.6 (10.4–17.4)	14.7 (11.2–18.9)	12.5 (9.9–16.1)	0.008^*∗*^
Night ACTH, pg/mL (IQR)	3.7 (1.0–5.0)	2.0 (1.0–5.0)	5.0 (2.0–6.0)	<0.001^*∗*^
Night cortisol, *µ*g/dL (IQR)	10.6 (6.7–17.0)	14.1 (9.4–18.6)	7.8 (5.7–12.3)	<0.001^*∗*^
1 mg DST cortisol, *µ*g/dL (IQR)	9.2 (5.0–16.6)	14.6 (7.4–19.4)	7.0 (3.9–12.3)	<0.001^*∗*^
1 mg DST cortisol >5.0 *μ*g/dl, *n* (%)	176 (74.3)	97 (86.6)	79 (63.2)	<0.001^*∗*^
24-hour urinary cortisol, *µ*g/day (IQR)	63 (35–177)	117 (42–295)	49 (30–94)	0.003^*∗*^
DHEA-S, *µ*g/dL (IQR)	25 (14–63)	23 (12–43)	33 (16–74)	0.005^*∗*^

^*∗*^All *P* values <0.05 were considered statistically significant. ACTH, adrenocorticotrophic hormone; BMI, body mass index; BMD, bone mineral density; DHEA-S, dehydroepiandrosterone-sulfate; DST, overnight dexamethasone suppression test; IQR; interquartile range; OP; osteopenia; OS, osteoporosis; sCr; serum creatinine; YAM, young adult mean.

**Table 2:** Adjusted odds ratio for predicting osteoporosis and osteopenia in patients with adrenal adenoma associated with cortisol excess.

Model	Total (*n* = 237)			MACS (*n* = 141)	
	Model 1 odds ratio (95% CI) *P* value	Model 2 odds ratio (95% CI) *P* value	Model 3 odds ratio (95% CI) *P* value	Model 1 odds ratio (95% CI) *P* value	Model 2 odds ratio (95% CI) *P* value
Cortisol excess^a^	3.358 (1.630–6.917) <0.001^*∗*^	1.124 (1.070–1.181) <0.001^*∗*^	3.924 (2.045–7.530) <0.001^*∗*^	2.187 (0.973–4.914) 0.058	1.156 (1.046–1.278) 0.005^*∗*^
Age, years	1.008 (0.987–1.030) 0.461	1.024 (1.000–1.049) 0.053	1.019 (0.996–1.043) 0.113	1.036 (1.001–1.072) 0.042^*∗*^	1.041 (1.005–1.078) 0.024^*∗*^
Sex female	3.350 (1.526–7.356) 0.003^*∗*^	3.918 (1.726–8.894) 0.001^*∗*^	3.461 (1.541–7.772) 0.003^*∗*^	4.264 (1.473–12.340) 0.007^*∗*^	5.231 (1.730–15.814) 0.003^*∗*^
BMI, kg/m^2^	0.947 (0.890–1.007) 0.083	0.941 (0.882–1.003) 0.063	0.937 (0.878–0.999) 0.047^*∗*^	0.970 (0.897–1.049) 0.443	0.959 (0.884–1.041) 0.315
sCr, mg/dL	1.199 (0.780–1.843) 0.408	1.158 (0.734–1.828) 0.529	1.177 (0.744–1.862) 0.486	1.113 (0.608–2.037) 0.729	1.030 (0.511–2.077) 0.934

^a^Cortisol excess was evaluated by positivity of 1 mg DST cortisol level >5.0 *µ*g/dL (vs. 1 mg DST cortisol level of 1.9–5.0 *μ*g/dL) in model 1, continuous variables of 1 mg DST cortisol levels (*µ*g/dL) in model 2, and the presence of Cushingoid features in model 3. Values were adjusted by age, female sex, BMI, and sCr. ^*∗*^All *P* values < 0.05 were considered statistically significant. BMI, body mass index; CI, confidential interval; DST, overnight dexamethasone suppression test; MACS, mild autonomous cortisol secretion; sCr, serum creatinine.

**Table 3:** Unadjusted odds ratio of associating factors for predicting osteoporosis and osteopenia in male and female patients.

	Total		MACS	
	Men (*n* = 55) odds ratio (95% CI) *P* value	Women (*n* = 182) odds ratio (95% CI) *P* value	Men (*n* = 42) odds ratio (95% CI) *P* value	Women (*n* = 99) odds ratio (95% CI) *P* value
1 mg DST cortisol > 5.0 *μ*g/dl	16.000 (1.902–134.580) 0.003^*∗*^	2.190 (1.038–4.619) 0.041^*∗*^	11.077 (1.201–102.193) 0.013^*∗*^	1.340 (0.583–3.084) 0.533
1 mg DST cortisol, *μ*g/dl	1.150 (1.039–1.273) 0.007^*∗*^	1.092 (1.043–1.144) <0.001^*∗*^	1.221 (1.027–1.452) 0.032^*∗*^	1.092 (0.983–1.213) 0.100
Cushingoid features	6.343 (1.509–26.656) 0.014^*∗*^	2.701 (1.472–4.958) 0.002^*∗*^	ND	ND
Age, years	0.993 (0.947–1.041) 0.771	1.004 (0.983–1.026) 0.705	1.009 (0.949–1.073) 0.784	1.044 (1.006–1.084) 0.024^*∗*^
BMI, kg/m^2^	0.948 (0.816–1.101) 0.484	0.946 (0.886–1.011) 0.103	0.945 (0.771–1.159) 0.588	0.974 (0.898–1.058) 0.537

^*∗*^All *P* values <0.05 were considered statistically significant. BMI,, body mass index; CI, confidential interval; DST, overnight dexamethasone suppression test; MACS, mild autonomous cortisol secretion; ND, not determined.

**Table 4:** Adjusted odds ratio of associating factors for predicting osteoporosis and osteopenia in female patients.

Model	Model 1 Odds ratio (95% CI) *P* value		Model 2 Odds ratio (95% CI) *P* value		Model 3 Odds ratio (95% CI) *P* value
	Total (*n* = 182)	MACS (*n* = 99)	Total cases (*n* = 182)	MACS (*n* = 99)	Total (*n* = 182)
Cortisol excess^a^	2.561 (1.142–5.742) 0.022^*∗*^	1.686 (0.687–4.140) 0.254	1.124 (1.063–1.189) <0.001^*∗*^	1.129 (1.005–1.267) 0.041^*∗*^	3.833 (1.829–8.030) <0.001^*∗*^
Age, years.	1.010 (0.987–1.035) 0.386	1.041 (1.002–1.083) 0.041^*∗*^	1.031 (1.004–1.059) 0.027^*∗*^	1.047 (1.006–1.090) 0.024^*∗*^	1.025 (0.998–1.052) 0.067
BMI, kg/m^2^	0.947 (0.886–1.012) 0.109	0.972 (0.894–1.057) 0.508	0.941 (0.877–1.009) 0.086	0.963 (0.884–1.049) 0.390	0.931 (0.867–1.000) 0.051

^a^Cortisol excess was evaluated by positivity of 1 mg DST cortisol level >5.0 *µ*g/dL (vs. 1 mg DST cortisol level of 1.9–5.0 *μ*g/dL) in model 1, continuous valuables of 1 mg DST cortisol (*µ*g/dL) in model 2, and the presence of cushingoid features in model 3. Values were adjusted by age and BMI. ^*∗*^All *P* values <0.05 were considered statistically significant. BMI, body mass index; CI, confidential interval; DST, overnight dexamethasone suppression test; MACS, mild autonomous cortisol secretion.

**Table 5:** Receiver operator characteristic curve analysis for predicting osteoporosis and osteopenia by continuous valuables of 1 mg dexamethasone suppression test cortisol levels.

	Sex	Optimal cutoff value, *μ*g/dl (Sen, Spe)	AUC ± SE (95% CI)	*P* value
Total	Men (*n* = 55)	9.4 (0.692, 0.762)	0.780 ± 0.069 (0.644–0.916)	<0.001^*∗*^
	Women (*n* = 182)	12.6 (0.586, 0.771)	0.665 ± 0.041 (0.585–0.745)	<0.001^*∗*^
MACS	Men (*n* = 42)	7.5 (0.857, 0.811)	0.801 ± 0.098 (0.610–0.992)	0.012^*∗*^
	Women (*n* = 99)	6.5 (0.535, 0.571)	0.580 ± 0.058 (0.466–0.693)	0.170

^*∗*^All *P* values <0.05 were considered statistically significant. AUC, area under the curve; CI, confidential interval; MACS, mild autonomous cortisol secretion; SE, standard error; Sen, sensitivity; Spe, specificity.

## Data Availability

Some or all datasets generated during and/or analyzed during the current study are not publicly available but are available from the corresponding author on reasonable request.
